# Characteristics, Treatment, and Prognosis of Heart Failure Patients with Chronic Obstructive Pulmonary Disease in the Colombian Heart Failure Registry (RECOLFACA)

**DOI:** 10.3390/jcdd11090265

**Published:** 2024-08-28

**Authors:** Jorge Alberto Sandoval-Luna, Alex Rivera-Toquica, Clara Saldarriaga, Erika Martínez-Carreño, Alexis Llamas, Gustavo Adolfo Moreno-Silgado, Julián Vanegas-Eljach, Nelson Eduardo Murillo-Benítez, Ricardo Gómez-Palau, Carlos Andrés Arias-Barrera, Fernán Mendoza-Beltrán, Diego Hernán Hoyos-Ballesteros, Carlos Andrés Plata-Mosquera, Luis Eduardo Echeverría, Juan Esteban Gómez-Mesa

**Affiliations:** 1 Department of Cardiology, Cardiología siglo XXI, Ibagué 730001, Colombia; jorgesandovalcardiologo@hotmail.com; 2 Department of Cardiology, Centro Médico Para el Corazón, Pereira 660000, Colombia; alexrivera17@gmail.com; 3 Department of Cardiology, Clínica los Rosales, Pereira 660002, Colombia; 4 Department of Cardiology, Universidad Tecnológica de Pereira, Pereira 660003, Colombia; 5 Department of Cardiology, Clínica Cardio VID, Medellín 050036, Colombia; clarais@une.net.co; 6 Department of Cardiology, Institución Clínica Iberoamérica Sanitas, Barranquilla 080001, Colombia; erikammc@hotmail.com; 7 Department of Cardiology, Clínica Las Américas, Medellín 050030, Colombia; allaji.llamas@gmail.com; 8 Department of Cardiology, Hospicardio, Montería 230001, Colombia; guamosi@hotmail.com; 9 Department of Cardiology, Hospital Alma Máter, Medellín 050004, Colombia; julivanel@hotmail.com; 10 Department of Cardiology, Angiografía de Occidente, Cali 760044, Colombia; nemurillob@gmail.com; 11 Department of Cardiology, Clínica Imbanaco, Cali 760042, Colombia; ricardopalau10@yahoo.com; 12 Department of Cardiology, Clínica Universitaria Colombia, Bogotá 111711, Colombia; carlosandresmd@hotmail.com; 13 Department of Cardiology, Fundación Clínica Shaio, Bogotá 111711, Colombia; fernan.mendoza@shaio.org; 14 Department of Cardiology, Centro Cardiovascular & Diabetes Massalud, Armenia 630001, Colombia; massaludsas@gmail.com; 15 Department of Cardiology, Hospital Universitario Erasmo Meoz, Cúcuta 540003, Colombia; gustavito207@hotmail.com; 16 Department of Cardiology, Fundación Cardiovascular de Colombia, Floridablanca 681001, Colombia; luisedo10@gmail.com; 17 Department of Cardiology, Fundación Valle del Lili, Cali 760032, Colombia; 18 Department of Health Sciences, Universidad Icesi, Cali 760031, Colombia

**Keywords:** heart failure, pulmonary disease, chronic obstructive, comorbidity, mortality

## Abstract

The impact of chronic obstructive pulmonary disease (COPD) on heart failure (HF) has yet to be well assessed in the Latin American population. This study aimed to analyze the impact of COPD on HF patients from the Colombian Heart Failure Registry (RECOLFACA). The primary outcome was all-cause mortality. A Cox proportional-hazards regression model was used to assess the impact of COPD on mortality. From the total of 2528 patients in the registry, 2514 patients had information regarding COPD diagnosis. COPD diagnosis was associated with a worse functional class and higher use of diuretics than non-COPD patients (*p* < 0.001). Patients with this comorbidity had a significantly better ejection fraction (median ejection fraction 35% vs. 32% in non-COPD patients; *p* = 0.004), with a higher occurrence of HF with preserved ejection fraction (HFpEF) in the COPD group (*p* = 0.000). Finally, patients with COPD had a significantly higher risk of mortality in the multivariate regression model (HR 1.47; 95% CI 1.02, 2.11). COPD is a prevalent comorbidity among patients with HF in Colombia, showing a different clinical profile and a worse functional class than patients without this condition. Patients with COPD and HFpEF have a high mortality risk according to our results.

## 1. Introduction

Heart failure (HF) and chronic obstructive pulmonary disease (COPD) are highly prevalent disease entities with increasing global prevalence and share a common risk factor profile. While HF affects worldwide around 26 million individuals and is present in approximately one of every 100 individuals over 65 years [1,2], COPD is predicted to prevail in more than 300 million individuals worldwide, representing an overall prevalence of 11.7% (95% confidence interval [CI] 7.3%–14.0%) in the general population [3,4]. These two prevalent conditions frequently coexist due to common pathophysiological mechanisms [5,6]. In fact, it is estimated that 10% to 40% of patients with HF have COPD as well [7]. Among the common risk factors that these two diseases share are age, environmental pollution, smoking, inflammation, and oxidative stress [8]. This coexistence is related to high morbidity and mortality in the affected individuals and a high burden for healthcare systems worldwide [9].

Although evidence exists considering the effect of COPD on mortality in HF patients, little evidence is available in the Latin American population. Moreover, more data should be given regarding this region’s ethnographic, medical, and biochemical features of COPD and HF patients. Our study aimed to estimate the influence of COPD on the prognosis of HF patients that belong to the Colombian Heart Failure Registry (RECOLFACA).

## 2. Materials and Methods

### 2.1. Study Design and Population

RECOLFACA is a prospective cohort study that involved 60 healthcare facilities in Colombia. Patient registration initiated in February 2017 and finished in October 2019. Including criteria were adult (18 or older) ambulatory patients clinically diagnosed with HF that had one or more hospital admissions due to HF within the 12 months preceding enrollment. Patients with a history of heart transplant, patients with previous implantation of a ventricular assist device, or patients with the inability to comply with scheduled follow-ups (telephone or in-person) due to social or personal conditions were excluded from the study. Additionally, patients with neurological limitations or socioeconomic barriers (e.g., limited access to healthcare facilities, lack of family or social support) that could potentially affect treatment adherence and follow-up monitoring were also excluded from the study. The assessment of inclusion and exclusion criteria was performed by the treating physicians and research staff at each participating center during the initial screening and enrollment process. Detailed specific criteria for inclusion and exclusion and complementary methodological characteristics of the registry are provided elsewhere [10,11]. This research received approval from the Ethics committee of the Fundación Valle del Lili under the act number 174-2017.

### 2.2. Data Collection

Data collected included sociodemographic (sex, age, area of residence, and medical insurance), clinical (comorbidities, NYHA classification, pharmacological treatments, systolic blood pressure, heart rate, diastolic diameter of left ventricle (DDLV), Left Ventricular Ejection Fraction (LVEF), HF phenotype, prolonged QRS interval, QoL variables, and all-cause mortality), and physiological variables (hemoglobin, NT-proBNP, serum creatinine, GFR, hyperkalemia, hyponatremia) and were registered at baseline. All data regarding demographic, health-related, and laboratory variables were collected from the patient’s clinical records registered by each center independently on a registration platform designed for this study and through an electronic transfer format on the web. The comorbidities evaluated were chronic kidney disease, defined as an estimated glomerular filtration rate (eGFR) of less than 60 mL/min/1.73 m^2^, arterial hypertension (systolic blood pressure ≥ 140 mmHg or diastolic blood pressure ≥ 90 mmHg), atrial fibrillation (diagnosed based on a 12-lead ECG or the documented history of this condition), diabetes mellitus, COPD, thyroid disease, and dyslipidemia (defined as an elevated total cholesterol [≥200 mg/dL] or low-density lipoprotein [LDL] cholesterol [≥100 mg/dL] levels, or triglycerides ≥ 150 mg/dL, or currently receiving lipid-lowering medications). Regarding the pharmacology treatment, we evaluated the use of triple therapy, defined as the presence of the prescription for an angiotensin-converting enzyme inhibitor (ACEI) or angiotensin receptor blocker (ARB) or angiotensin receptor neprilysin inhibitor (ARNI), plus a beta-blocker plus a mineralocorticoid receptor antagonist (MRA), or other medicaments such as ivabradin, nitrates, antiplatelets, statins, and anticoagulants.

Data for COPD diagnosis, treatment, and positive spirometry test were taken from the report in the medical history. HF severity was classified with the New York Heart Association (NYHA) system. Patients’ QoL was evaluated at baseline using the EQ-5D questionnaire (available in Spanish). Those patients with LVEF ≥50% were categorized as HFpEF, patients with LVEF between 41 and 49% were classified as HFmrEF, and patients with LVEF ≤40% were considered as HFrEF. Moreover, an ischemic disease diagnosis was recorded if the individual experienced a coronary revascularization technique or presented a previous myocardial infarction history. All the comorbidities assessed are described elsewhere [10,11]. Some patients presented supplementary echocardiographic variables, such as the left ventricle ejection fraction (LVEF) and the left ventricle systolic diameter, including others.

### 2.3. Outcomes

The main variable evaluated was all-cause mortality. Information regarding this outcome was gathered through a questionnaire filled out by each HF clinic and center at the ≥6-month follow-up.

### 2.4. Statistical Analysis

The total sample was divided into two groups (COPD vs. non-COPD patients). Continuous variables were described using medians and interquartile ranges (IQRs), while categorical variables were recorded as absolute counts, percentages, and proportions. To measure the disparities between groups, categorical variables were evaluated using Pearson’s chi-squared and Fisher’s exact tests, while continuous variables were evaluated using Mann–Whitney U tests. The cumulative incidence of mortality was estimated with 95% confidence intervals (CIs). A Cox proportional-hazards regression model incorporating multiple variables was employed to identify the prognostic significance of having a COPD diagnosis. A *p*-value less than 0.05 (determined through a two-tailed test) was considered statistically significant. All tests were made using Statistical Package STATA version 15 (Station College, TX, USA).

## 3. Results

Between 2017 and 2019, RECOLFACA enrolled 2528 patients, and from those, 2514 patients had information concerning COPD diagnosis. COPD prevalence among these patients was 17.5% (n = 441).

### 3.1. Sociodemographic Factors and Comorbidities

Patients diagnosed with COPD were significantly older (median age: 76 vs. 68 years, *p* < 0.001), while no significant distinctions by sex were detected (*p* = 0.142). Compared to non-COPD patients, the higher median number of comorbidities was found in COPD patients (*p* < 0.001), mainly chronic kidney disease, thyroid disease, atrial fibrillation, and hypertension (Table 1). The most common HF phenotype for both groups was HFrEF, followed by HFmrEF and HFpEF; however, while HFrEF was significantly more common in the non-COPD group (*p* = 0.000), in the COPD group, HFpEF was significantly more common (*p* = 0.000).

### 3.2. Clinical, Laboratory, and Echocardiographic Differences

COPD patients had a significantly inferior NYHA functional class, with a higher number of patients in class III or IV (46.7% vs. 31.8%. *p* < 0.001). Moreover, they had a significantly higher median systolic blood pressure (*p* < 0.001) while reporting a lower median quality of life score (EQ-5D) compared to non-COPD patients (*p* < 0.001). Furthermore, COPD patients present significantly worse eGFR (*p* = 0.007) and higher serum creatinine (*p* = 0.012) than patients without this condition. Following the worse NYHA functional class, individuals diagnosed with COPD presented a significantly higher median value of N-terminal pro-brain natriuretic peptide (NT-proBNP) (3523 vs. 2132, respectively. *p* = 0.046). Finally, no significant differences concerning implantable devices between both groups were observed (Table 1).

Regarding echocardiographic variables, patients diagnosed with COPD presented a significantly higher median LVEF related to the non-COPD group (median LVEF 35% vs. 32%. *p* = 0.004) (Table 1); these results reflected a higher occurrence of HF with preserved ejection fraction (HFpEF) in the COPD group (*p* = 0.000). Similarly, COPD patients had a significantly lower diastolic diameter of the left ventricle (*p* = 0.037) while reporting a higher rate of pulmonary hypertension (*p* < 0.001).

### 3.3. Heart Failure Treatment

Pharmacological prescription patterns were similar for almost all HF medications reported in the RECOLFACA among the two groups. Although beta-blockers and antiplatelets were the most prescribed pharmacological treatment for both groups, and ivabradine was the least prescribed, COPD patients were significantly more regularly prescribed diuretics (75.7% vs. 65.6%. *p* < 0.001) and less regularly prescribed ivabradine (3.4% vs. 6.5%. *p* = 0.012) than non-COPD patients. When analyzing these two medicaments separately by HF phenotype, we found that diuretics in the COPD group were significantly less prescribed to patients with HFrEF (*p* = 0.003) and more prescribed to patients with HFpEF (*p* = 0.012) (Table 1). Regarding ivabradine, it was significantly less prescribed to patients with HFrEF (*p* = 0.006).

### 3.4. Mortality

Median follow-up period was 215 days (Q1: 188; Q3: 254), and 6.76% of the patients died during the follow-up period, showing a mortality rate of 0.29 per 1000 person-years (95% CI 25.4–34.5). We observed a significantly higher mortality rate in the COPD group (rate per 1000 person-time: 0.45 vs. 0.27, respectively; *p* = 0.002). Finally, after adjusting for sex, age, smoking, hypertension, chronic kidney disease, and diuretic use in the multivariate regression model, patients with COPD presented a significantly higher risk of mortality (HR 1.47; 95% CI 1.02, 2.11).

## 4. Discussion

In the present study, we observed a prevalence of COPD in HF patients of 17.5%, highlighting an older age and a more significant number of comorbidities, mainly chronic kidney disease, thyroid disease, atrial fibrillation, and arterial hypertension. Furthermore, patients with COPD had a significantly worse NYHA functional class and elevated NT-proBNP levels. They were more frequently prescribed diuretics despite showing a superior prevalence of HFpEF and lower ventricular diameters. Finally, COPD diagnosis was significantly related to higher mortality risk after adjustment for relevant covariates. The difference in mortality may be explained by a greater severity of HF but may also be related to the association with lung disease, which is associated with higher mortality. Both findings can be related to more severe HF and higher NT-proBNP levels.

Several studies have considered the prevalence of COPD diagnosis in HF patients utilizing community-based studies and institutional registries. Most of them were performed in developed countries, reporting prevalence ranging from 10% to 25%. Nevertheless, to our knowledge, this represents one of the first studies of this type in Colombia. The coexistence of these two conditions is related to the common pathophysiological pathways present in COPD and most cardiovascular diseases. For example, several studies have observed that low-grade systemic inflammation, such as the one present even in stable COPD and moderate or severe airflow obstruction, can significantly increase the probability to develop ischemic heart disease [8].

Markers of cardiovascular illness such as subclinical atherosclerosis, arterial stiffness, and endothelial dysfunction have been strongly associated with airflow limitation. In this context, inflammation has a central participation in the common pathogenesis of HF and COPD [12], as systemic inflammation favors the progression of coronary atherosclerosis, promoting the development of ischemic heart illness, the most common origin of HF worldwide. Nonetheless, we did not observe a significant alteration in the prevalence of coronary heart disease among the two groups, a result that has also been described in similar cohorts worldwide [13,14,15].

Pulmonary hypertension secondary to severe COPD can play a relevant function in the progress of HF in the absence of coronary artery disease. Multiple studies have reported that COPD patients have a higher probability of developing left ventricular diastolic dysfunction in comparison to those without COPD [16,17,18]. Moreover, it has been reported that COPD is more prevalent in patients with the HFpEF phenotype compared to other types [19]. According to the Euro Heart Failure survey [20], the prevalence of COPD in hospital cohorts of the HFpEF population from multiple European countries was between 14 and 34%. In the study by Adeel et al. [21], performed in the United States, a statistically significant higher number of patients with HFpEF and concurrent COPD (33.5% compared to 25%) was also reported. These results are in line with our result of a higher prevalence of HFpEF in the COPD group (26.9% in COPD group vs. 14.6% in non-COPD group). The explanation for this could be associated with the similar physiopathological mechanisms shared between HFpEF and COPD (i.e., systemic inflammation and increased oxidative stress) [22]. Our results indicating that most patients had the HFrEF phenotype in the COPD group contrast with a recent published study by Gulea et al. [23] that analyzed data from American patients where they found in a COPD group that most of them presented the HFpEF phenotype (20% had HFrEF, 10% had HFmEF, and 70% had HFpEF).

On the other hand, the observation of a worse NYHA functional class in COPD patients could be attributed to a potential overestimation of the clinical evaluation of the NYHA functional class in these patients [24]. This phenomenon is derived from the poorer exercise tolerance resulting from functional lung impairment in COPD patients, making it challenging for the clinician to differentiate the effects of COPD and HF when evaluating symptoms in these populations [25].

We identified a significantly high rate of diuretic use in COPD patients, which has been reported in other studies and may reflect an increased volume overload status derived from pulmonary hypertension, right-sided HF, and concomitant steroid use [13,15,26,27,28].

Notably, the higher mortality risk here observed in COPD patients has been reported in other studies published in the literature [13,14,26,28,29]. The reasoning behind these findings is multifactorial, for instance, a higher susceptibility to lung and systemic infections and increasing the risk of HF decompensation. On the other hand, low-grade chronic inflammation may play a role in developing new cardiovascular events, such as stroke and myocardial infarction [30]. Right-sided ventricular dysfunction, a relatively frequent finding in severe COPD patients, is highly correlated with NYHA functional class and has been identified as a marker of mortality in HF patients [31]. Finally, COPD patients frequently exhibit a high degree of frailty, significantly adding to the already high burden of HF [32,33]. On the other hand, treatment-related factors may play a central function in the diminished prognosis detected in patients with concomitant HF and COPD [13]. Specifically, both diseases could promote suboptimal therapy, while beta-adrenergic agonists could raise the risk of unfavorable cardiac events, and the non-selective beta adrenergic blockade could induce bronchoconstriction [34,35]. This could significantly impact the prescription rate of important HF medicaments in COPD patients. However, our results suggested a similar use of HF medications in both groups.

### Study Limitations

The RECOLFACA did not incorporate specific or detailed data concerning COPD severity and treatment, limiting the opportunity of incorporating these significant factors into the analyses. Also, several potential confounders were not accounted for in the present study, such as the body mass index or other relevant diagnoses (e.g., asthma). We aimed to address this limitation by incorporating demographic variables, medical history, laboratory test results, echocardiographic tests, and device therapy details into the study. We did not count data on echocardiographic variables from all patients, which could be a confounding factor. Therefore, our results should be interpreted with caution.

## 5. Conclusions

COPD is a predominant comorbidity in patients with HF, highlighting a distinctive clinical profile and an inferior NYHA functional class compared to non-COPD patients. According to our findings, patients with COPD and HFpEF have a higher mortality risk. A multidisciplinary approach to these patients, including internists, pulmonologists, and respiratory therapists, could positively impact the course of both of these pathologies when presented combined. Further prospective cohort studies that include data regarding the severity of lung dysfunction and that include in the analysis body mass index (BMI) and other relevant diagnoses such as asthma are required to validate our results in this population.

## Figures and Tables

**Table 1:** Sociodemographic and clinical characteristics by COPD diagnosis.

	Non-COPD (n = 2073)	COPD (n = 441)	Total (n = 2514)	*p*-Value
Age, years [Median(IQR)]	68 (58, 76)	76 (70, 82)	69 (59, 78)	<0.001
Sex, males	1207 (58.2%)	240 (54.4%)	1447 (57.6%)	0.142
Population				0.542
Asian	1 (0.0%)	0 (0.0%)	1 (0.0%)	
European	88 (4.2%)	28 (6.3%)	116 (4.6%)	
Indigenous	8 (0.4%)	2 (0.5%)	10 (0.4%)	
Hispanic	1532 (73.9%)	321 (72.8%)	1853 (73.7%)	
Mestiza	380 (18.3%)	78 (17.7%)	458 (18.2%)	
African American	64 (3.1%)	12 (2.7%)	76 (3.0%)	
Insurance				0.050
Private/Contributive	1235 (59.6%)	235 (53.3%)	1470 (58.5%)	
Additional policy	119 (5.7%)	28 (6.3%)	147 (5.8%)	
Public/Subsidized	719 (34.7%)	178 (40.4%)	897 (35.7%)	
Arterial hypertension	1443 (69.6%)	368 (83.4%)	1811 (72.0%)	<0.001
Type 2 diabetes mellitus	507 (24.5%)	113 (25.6%)	620 (24.7%)	0.606
Cancer	86 (4.1%)	15 (3.4%)	101 (4.0%)	0.468
Liver disease	10 (0.5%)	1 (0.2%)	11 (0.4%)	0.460
Depression	39 (1.9%)	8 (1.8%)	47 (1.9%)	0.925
Coronary heart disease	590 (28.5%)	116 (26.3%)	706 (28.1%)	0.360
Atrial fibrillation	434 (20.9%)	126 (28.6%)	560 (22.3%)	<0.001
Thyroid disease	298 (14.4%)	90 (20.4%)	388 (15.4%)	0.001
Chronic kidney disease	324 (15.6%)	110 (24.9%)	434 (17.3%)	<0.001
Valvulopathy	359 (17.3%)	70 (15.9%)	429 (17.1%)	0.464
CABG	143 (6.9%)	27 (6.1%)	170 (6.8%)	0.556
Dyslipidemia	534 (25.8%)	113 (25.6%)	647 (25.7%)	0.953
Smoking	324 (15.6%)	128 (29.0%)	452 (18.0%)	<0.001
Mean number of comorbidities	2 (1, 3)	4 (3, 5)	3 (2, 4)	<0.001
NYHA functional class				<0.001
I	277 (13.4%)	21 (4.8%)	298 (11.9%)	
II	1136 (54.8%)	214 (48.5%)	1350 (53.7%)	
III	574 (27.7%)	173 (39.2%)	747 (29.7%)	
IV	86 (4.1%)	33 (7.5%)	119 (4.7%)	
HF phenotypes (Non COPD n = 347; COPD n = 1694)				
HFrEF	1273 (75.1%)	227 (65.4%)	1500 (73.5%)	0.000
HFmrEF	174 (10.3%)	37 (10.7%)	211 (10.3%)	0.827
HFpEF	247 (14.6%)	83 (23.9%)	330 (16.2%)	0.000
ACEI/ARB	1541 (74.3%)	339 (76.9%)	1880 (74.8%)	0.266
HFrEF	936 (73.9%)	172 (63.5%)	1108 (72.1%)	0.478
HFmrEF	137 (10.8%)	30 (11.1%)	167 (10.9%)	0.750
HFpEF	192 (15.2%)	69 (25.5%)	266 (17.3%)	0.501
Diuretics	1359 (65.6%)	334 (75.7%)	1693 (67.3%)	<0.001
HFrEF	874 (78.9%)	178 (67.4%)	1052 (76.7%)	0.003
HFmrEF	97 (8.8%)	27 (10.2%)	124 (9.0%)	0.053
HFpEF	137 (12.4%)	59 (22.3%)	196 (14.3%)	0.012
Beta-blockers	1806 (87.1%)	383 (86.8%)	2189 (87.1%)	0.877
HFrEF	1130 (77.3%)	200 (67.1%)	1330 (75.6%)	0.772
HFmrEF	143 (9.8%)	33 (11.1%)	176 (10.0%)	0.298
HFpEF	189 (12.9%)	65 (21.8%)	254 (14.4%)	0.737
ARNI	210 (10.1%)	35 (7.9%)	245 (9.7%)	0.158
HFrEF	162 (93.1%)	24 (85.7%)	186 (92.1%)	0.365
HFmrEF	9 (5.2%)	3 (10.7%)	12 (5.9%)	0.588
HFpEF	3 (1.7%)	1 (3.6%)	4 (2.0%)	0.994
MRA	1168 (56.3%)	231 (52.4%)	1399 (55.6%)	0.128
HFrEF	835 (86.6%)	159 (85.9%)	994 (86.5%)	0.191
HFmrEF	69 (7.2%)	11 (5.9%)	80 (6.9%)	0.258
HFpEF	60 (6.2%)	15 (8.1%)	75 (6.5%)	0.242
Ivabradine	135 (6.5%)	15 (3.4%)	150 (6.0%)	0.012
HFrEF	113 (91.9%)	8 (72.7%)	121 (90.3%)	0.006
HFmrEF	6 (4.9%)	3 (27.3%)	9 (6.7%)	0.203
HFpEF	4 (3.3%)	0 (0.0%)	4 (3.0%)	0.243
Antiplatelets	1643 (79.3%)	344 (78.0%)	1987 (79.0%)	0.557
HFrEF	596 (74.3%)	99 (63.9%)	695 (72.6%)	0.372
HFmrEF	89 (11.1%)	24 (15.5%)	113 (11.8%)	0.129
HFpEF	117 (14.6%)	32 (20.6%)	149 (15.6%)	0.163
Implantable devices				0.237
No	1924 (92.8%)	415 (94.1%)	2339 (93.0%)	
Resynchronization therapy	44 (2.1%)	4 (0.9%)	48 (1.9%)	
Resynchronization therapy + ICD	105 (5.1%)	22 (5.0%)	127 (5.1%)	
Systolic blood pressure (mmHg) [Median(IQR)]	120 (105, 132)	120 (110, 138)	120 (106, 134)	<0.001
Heart rate [Median(IQR)]	72 (65, 80)	72 (65, 81)	72 (65, 81)	0.871
Prolonged QRS duration	400 (36.7%)	103 (41.7%)	503 (37.6%)	0.146
Quality of life (EQ-5D) score [Median(IQR)]	85 (70, 100)	75 (60, 90)	80 (65, 100)	<0.001
LVEDD (mm) [Median(IQR)]	57 (48, 65)	55 (46, 64)	57 (48, 65)	0.037
LVEF (%) [Median(IQR)]	32 (25, 40)	35 (25, 47)	33 (25, 42)	0.004
Pulmonary hypertension	687 (45.2%)	203 (64.9%)	890 (48.6%)	<0.001
Hemoglobin (mg/dl) [Median(IQR)]	13 (11.7, 14.3)	13 (11.300, 14.2)	13 (11.6, 14.3)	0.364
Serum creatinine [Median(IQR)]	1.100 (0.9, 1.4)	1.190 (0.932, 1.5)	1.100 (0.9, 1.4)	0.012
GFR [Median(IQR)]	58.490 (43.2, 78.5)	53.405 (39.0, 71.7)	57.450 (42.6, 77.4)	0.007
Hyperkalemia	141 (9.2%)	35 (10.2%)	176 (9.4%)	0.581
Hyponatremia	196 (14.1%)	44 (14.0%)	240 (14.0%)	0.966
NT-proBNP [Median(IQR)]	2132 (873, 5498)	3523 (1225, 6600)	2255.500 (954, 5593.8)	0.046

COPD: chronic obstructive pulmonary disease; CABG: coronary artery bypass graft; ACEI: angiotensin-converting enzyme inhibitor; ARB: angiotensin receptor blockers; ARNI: angiotensin receptor neprilysin inhibitor; MRA: mineralocorticoid receptor antagonists; LVEDD: left ventricular end diastolic diameter; LVEF: left ventricular ejection fraction; GFR: glomerular filtration rate; NT-ProBNP: N-terminal pro-brain natriuretic peptide; IQR: interquartile range.

## Data Availability

The datasets used and/or analyzed during the current study are available from the corresponding author upon reasonable request.

## References

[1] Savarese G., Lund L.H. (2017). Global Public Health Burden of Heart Failure. Card. Fail. Rev..

[2] Virani S.S., Alonso A., Benjamin E.J., Bittencourt M.S., Callaway C.W., Carson A.P., Chamberlain A.M., Chang A.R., Cheng S., Delling F.N. (2020). Heart Disease and Stroke Statistics-2020 Update: A Report from the American Heart Association. Circulation.

[3] Adeloye D., Chua S., Lee C., Basquill C., Papana A., Theodoratou E., Nair H., Gasevic D., Sridhar D., Campbell H. (2015). Global and regional estimates of COPD prevalence: Systematic review and meta-analysis. J. Glob. Health.

[4] Quaderi S.A., Hurst J.R. (2018). The unmet global burden of COPD. Glob. Health Epidemiol. Genom..

[5] Cuthbert J.J., Pellicori P., Clark A.L. (2022). Optimal Management of Heart Failure and Chronic Obstructive Pulmonary Disease: Clinical Challenges. Int. J. Gen. Med..

[6] Wang A., Li Z., Sun Z., Liu Y., Zhang D., Ma X. (2023). Potential Mechanisms Between HF and COPD: New Insights from Bioinformatics. Curr. Probl. Cardiol..

[7] Santellano-Juárez B., González-Islas D., Contreras-Ramírez E., Orea-Tejeda A., Pineda-Juárez J., Peláez-Hernández V., Keirns-Davies C., Herrera-Saucedo R., Elizondo-Montes M., Pérez-Cortes G. (2017). Association between right heart failure and hospital mortality in exacerbation of COPD. Neumol. Cirugía Tórax.

[8] Onishi K. (2017). Total management of chronic obstructive pulmonary disease (COPD) as an independent risk factor for cardiovascular disease. J. Cardiol..

[9] Morgan A.D., Zakeri R., Quint J.K. (2018). Defining the relationship between COPD and CVD: What are the implications for clinical practice?. Ther. Adv. Respir. Dis..

[10] Gómez-Mesa J.E., Saldarriaga-Giraldo C.I., Echeverría L.E., Luna-Bonilla P., Grupo Investigador RECOLFACA (2021). Registro Colombiano de falla cardiaca (RECOLFACA): Resultados. Rev. Colomb. Cardiol..

[11] Gómez-Mesa J.E., Saldarriaga C.I., Echeverría L.E., Luna P., RECOLFACA Research Group (2021). Colombian heart failure registry (RECOLFACA): Methodology and preliminary data. Rev. Colomb. Cardiol..

[12] Schafauser N.S., Sampaio L.M.M., Heubel A.D., Kabbach E.Z., de Oliveira Kawakami D.M., Leonardi N.T., Castello-Simões V., Borghi-Silva A., Mendes R.G. (2024). Influence of heart failure (HF) comorbidity in chronic obstructive pulmonary disease (COPD) and isolated forms of HF and COPD on cardiovascular function during hospitalization. Respir. Med..

[13] Macchia A., Monte S., Romero M., D’Ettorre A., Tognoni G. (2007). The prognostic influence of chronic obstructive pulmonary disease in patients hospitalised for chronic heart failure. Eur. J. Heart Fail..

[14] De Blois J., Simard S., Atar D., Agewall S., Norwegian Heart Failure Registry (2010). COPD predicts mortality in HF: The Norwegian Heart Failure Registry. J. Card. Fail..

[15] Rusinaru D., Saaidi I., Godard S., Mahjoub H., Battle C., Tribouilloy C. (2008). Impact of chronic obstructive pulmonary disease on long-term outcome of patients hospitalized for heart failure. Am. J. Cardiol..

[16] Cherneva Z., Valev D., Youroukova V., Cherneva R. (2021). Left ventricular diastolic dysfunction in non-severe chronic obstructive pulmonary disease—A step forward in cardiovascular comorbidome. PLoS ONE.

[17] Boussuges A., Pinet C., Molenat F., Burnet H., Ambrosi P., Badier M., Sainty J.M., Orehek J. (2000). Left atrial and ventricular filling in chronic obstructive pulmonary disease. An echocardiographic and Doppler study. Am. J. Respir. Crit. Care Med..

[18] Louie E.K., Rich S., Levitsky S., Brundage B.H. (1992). Doppler echocardiographic demonstration of the differential effects of right ventricular pressure and volume overload on left ventricular geometry and filling. J. Am. Coll. Cardiol..

[19] Xu S., Gu Z., Zhu W., Feng S. (2024). Association of COPD with adverse outcomes in heart failure patients with preserved ejection fraction. ESC Heart Fail..

[20] Cleland J.G.F., Swedberg K., Follath F., Komajda M., Cohen-Solal A., Aguilar J.C., Dietz R., Gavazzi A., Hobbs R., Korewicki J. (2003). The EuroHeart Failure survey programme—A survey on the quality of care among patients with heart failure in Europe. Part 1: Patient characteristics and diagnosis. Eur. Heart J..

[21] Adeel M., Wakefield D., Soucier R., ZuWallack R., Tabtabai S. (2023). The Presence of Clinical COPD Predicts Mortality in Heart Failure Patient Population. J. Card. Fail..

[22] Khan S.S., Kalhan R. (2022). Comorbid Chronic Obstructive Pulmonary Disease and Heart Failure: Shared Risk Factors and Opportunities to Improve Outcomes. Ann. Am. Thorac. Soc..

[23] Gulea C., Zakeri R., Quint J.K. (2022). Differences in Outcomes between Heart Failure Phenotypes in Patients with Coexistent Chronic Obstructive Pulmonary Disease: A Cohort Study. Ann. Am. Thorac. Soc..

[24] Güder G., Rutten F.H., Brenner S., Angermann C.E., Berliner D., Ertl G., Jany B., Lammers J.W., Hoes A.W., Störk S. (2012). The impact of heart failure on the classification of COPD severity. J. Card. Fail.

[25] Mooney L., Hawkins N.M., Jhund P.S., Redfield M.M., Vaduganathan M., Desai A.S., Rouleau J.L., Minamisawa M., Shah A.M., Lefkowitz M.P. (2021). Impact of Chronic Obstructive Pulmonary Disease in Patients with Heart Failure with Preserved Ejection Fraction: Insights from PARAGON-HF. J. Am. Heart Assoc..

[26] Mentz R.J., Fiuzat M., Wojdyla D.M., Chiswell K., Gheorghiade M., Fonarow G.C., O’Connor C.M. (2012). Clinical characteristics and outcomes of hospitalized heart failure patients with systolic dysfunction and chronic obstructive pulmonary disease: Findings from OPTIMIZE-HF. Eur. J. Heart Fail..

[27] Iversen K.K., Kjaergaard J., Akkan D., Kober L., Torp-Pedersen C., Hassager C., Vestbo J., Kjoller E., ECHOS-Lung Function Study Group (2008). Chronic obstructive pulmonary disease in patients admitted with heart failure. J. Intern. Med..

[28] Fisher K.A., Stefan M.S., Darling C., Lessard D., Goldberg R.J. (2015). Impact of COPD on the mortality and treatment of patients hospitalized with acute decompensated heart failure: The Worcester Heart Failure Study. Chest.

[29] Sato Y., Yoshihisa A., Oikawa M., Nagai T., Yoshikawa T., Saito Y., Yamamoto K., Takeishi Y., Anzai T. (2019). Prognostic impact of chronic obstructive pulmonary disease on adverse prognosis in hospitalized heart failure patients with preserved ejection fraction—A report from the JASPER registry. J. Cardiol..

[30] Vasan R.S., Sullivan L.M., Roubenoff R., Dinarello C.A., Harris T., Benjamin E.J., Sawyer D.B., Levy D., Wilson P.W., D’Agostino R.B. (2003). Inflammatory markers and risk of heart failure in elderly subjects without prior myocardial infarction: The Framingham Heart Study. Circulation.

[31] Raina A., Meeran T. (2018). Right Ventricular Dysfunction and Its Contribution to Morbidity and Mortality in Left Ventricular Heart Failure. Curr. Heart Fail. Rep..

[32] Yee N., Locke E.R., Pike K.C., Chen Z., Lee J., Huang J.C., Nguyen H.Q., Fan V.S. (2020). Frailty in Chronic Obstructive Pulmonary Disease and Risk of Exacerbations and Hospitalizations. Int. J. Chron Obstruct. Pulmon Dis..

[33] Kennedy C.C., Novotny P.J., LeBrasseur N.K., Wise R.A., Sciurba F.C., Benzo R.P. (2019). Frailty and Clinical Outcomes in Chronic Obstructive Pulmonary Disease. Ann. Am. Thorac. Soc..

[34] Salpeter S.R., Ormiston T.M., Salpeter E.E. (2004). Cardiovascular effects of beta-agonists in patients with asthma and COPD: A meta-analysis. Chest.

[35] Sirak T.E., Jelic S., Le Jemtel T.H. (2004). Therapeutic update: Non-selective beta- and alpha-adrenergic blockade in patients with coexistent chronic obstructive pulmonary disease and chronic heart failure. J. Am. Coll. Cardiol..

